# Potatoes Compared with Rice in Meals with either Animal or Plant Protein Reduce Postprandial Glycemia and Increase Satiety in Healthy Adults: A Randomized Crossover Study

**DOI:** 10.1016/j.tjnut.2024.08.017

**Published:** 2024-08-23

**Authors:** Amira M Amr, G Harvey Anderson, Shirley Vien, Hrvoje Fabek

**Affiliations:** Department of Nutritional Sciences, Temerty Faculty of Medicine, University of Toronto, Medical Sciences Building, Toronto, Ontario, Canada

**Keywords:** glycemic control, blood glucose, potato, food intake, satiety, insulin, adults

## Abstract

**Background:**

Rice and pasta are recommended as healthier than potatoes on the basis of their glycemic index when eaten alone.

**Objectives:**

The study objective was to evaluate postprandial glycemia (PPG), appetite, and food intake (FI) at meals with potatoes or rice when consumed with either meatballs or their vegetarian substitute.

**Methods:**

In a randomized, single-blinded, crossover design, 26 (13 males and 13 females) healthy adults (age: 18–45 y; body mass index [kg/m^2^]: 18.5–29.9) consumed isocaloric fixed amounts of either meatballs or vegetarian-substitute balls with *ad libitum* access to either baked French fries (BFF), instant mashed potatoes (IMPs), or rice (control). FI was measured at the meal and at an *ad libitum* pizza meal served 120 min later. Blood glucose (BG), appetite, and plasma insulin responses were measured within the meal (0–30 min), postmeal (30–120 min), within pizza meal (120–140 min), and post-pizza (140–170 min). Effects of protein source, carbohydrate (CHO) source, and sex and their interactions were analyzed using analysis of variance followed by Tukey’s post hoc test.

**Results:**

Participants consumed 23–25% less treatment meal energy (kcal), 32–34% less CHO energy (kcal), and 13–16% less total energy (kcal) after the BFF and IMP than rice meals (*P* < 0.0001). Postmeal BG was lower after IMP (6.76 ± 0.15; *P* < 0.0001) and rice (6.92 ± 0.15; *P* = 0.0012) compared with BFF (7.19 ± 0.15). Post-pizza BG was higher after rice (6.77 ± 0.09) than that after BFF (6.51 ± 0.09; *P* = 0.0012) and IMP (6.39 ± 0.09; *P* < 0.0001). Postmeal meaned insulin was higher after BFF (82.16 ± 8.58) and IMP (77.75 ± 8.60) compared with rice (56.44 ± 8.59; *P* < 0.002). Insulin during pizza meal was lower after BFF (17.14 ± 6.90) compared with both IMP (39.03 ± 6.90; *P* = 0.0060) and rice (34.21 ± 6.90; *P* = 0.0336). Meatballs led to lower BG (6.48 ± 0.09; *P* = 0.0076) and higher insulin (84.54 ± 5.87; *P* = 0.0406) post-pizza compared with their plant protein substitute (6.64 ± 0.09 and 73.18 ± 5.87, respectively).

**Conclusions:**

Adults consuming meatballs or plant-based substitute with *ad libitum* IMP had lower PPG post-treatment and at a later pizza meal compared with rice. Both IMP and BFF resulted in lower energy intake than after rice.

This trial was registered at http://clinicaltrials.gov (https://register.clinicaltrials.gov/prs/app/action/SelectProtocol?sid=S000CKIJ&selectaction=Edit&uid=U0000IA4&ts=2&cx=-uf51kf) as NCT05610124. Protocol ID: 43406 (Postprandial Glycemia and Satiety of Meals with Potatoes, with and without Protein).

## Introduction

Potatoes have been a staple food for centuries; however, recent dietary recommendations discourage the consumption of white vegetables, including potatoes. This guidance overlooks the fact that potatoes are rich in essential nutrients, including fiber, vitamins (B6, folate, C, E, and K), and minerals (potassium, iron, magnesium, and copper) [1,2]. Despite their low energy density and balanced nutritional profile [3], potato consumption has declined over the past several decades [4]. This decline is partly because of the concerns about their high glycemic index (GI) when consumed in isolation [5]. However, recent evidence challenges this perception, emphasizing that potatoes are predominantly consumed as part of a meal. Therefore, to accurately assess their impact on postprandial glycemia (PPG) and caloric intake, it is important to evaluate potatoes within the context of a complete meal rather than in isolation [3].

Some epidemiological studies have reported positive associations between potato intake and obesity, type 2 diabetes mellitus, cardiovascular diseases, and characteristics of metabolic syndrome. However, the evidence is weak, and the perceived association with obesity is primarily linked to the frequent consumption of quick-service restaurant foods, where deep-fried potatoes are commonly served [5]. Potatoes are consumed in various forms at meals, and their nutrient content is significantly influenced by cooking methods. For instance, boiling potatoes in water can cause the leaching of water-soluble nutrients, whereas frying in oil can increase the resistant starch content of the cooked potatoes [2]. Surprisingly, there is a scarcity of studies examining the impact of cooking methods and the quantity of potatoes consumed, relative to other starchy carbohydrates (CHO), on PPG, satiety, and subsequent food intake (FI).

Potatoes, as a group, exhibit a broad GI range from 35 to 103, reflecting the diversity of potato varieties and the effects of different cooking and processing methods. Mean reported GI values include 84 for instant mashed potatoes (IMPs), 79 for regular mashed potatoes, 73 for boiled potatoes, 49 for cooked potatoes that were refrigerated overnight [6] and 77 for French fries [7]. Potatoes have a high satiety index (323%) and are more satiating than other starchy CHO, such as pasta (GI of 52%), rice (GI of 67%) [2,6], and white bread (GI of 100%) [8]. This underscores the need for a more nuanced understanding of the role of CHO in meals, considering not only their GI but also their satiety-inducing properties in various forms and preparations.

Predicting the energy intake and glycemic responses of meals containing CHO and protein sources based solely on their individual GI values has been shown to be inadequate [[9], [10], [11], [12], [13]]. Although limited measures of metabolic regulatory hormones have been reported, existing data suggest that the physiologic interaction of CHO and protein foods in a meal collaboratively reduces appetite and PPG. For instance, the swift digestion of mashed potatoes results in a rapid increase in blood glucose (BG), followed by a sharp and sustained decrease in appetite because of the satiety signals from both glucose and protein, as observed in our prior study involving children [9].

Potatoes are typically consumed alongside animal-based protein sources in meals. However, contemporary dietary guidelines advocate for increased consumption of plant-based proteins as alternatives to animal-derived foods. This shift has led to the development and increased use of plant-based substitutes for many animal products. Consequently, there is a growing trend toward vegetarian dietary practices that incorporate plant-based substitutes in meals. This trend presents an opportunity to include potatoes, in various forms, as well as other staples, such as rice or pasta, in these meals.

Therefore, the objective of this study was to investigate the effects of potato consumption served with either meat or vegetarian “meat” substitute on mealtime FI and BG as the primary measures, as well as on insulin, satiety, and FI at a later meal in normal-weight healthy adults. We simulated meals that might be eaten at home, feeding adult participants meals with a fixed amount of protein from either beef or a vegetarian substitute, along with *ad libitum* access to IMP, baked French fries (BFF), or white rice as a comparator. We hypothesized that potatoes in 2 common forms (IMP or BFF), when consumed with either animal or vegetarian protein sources, would result in reduced appetite and PPG, ultimately leading to lower FI during a subsequent meal compared with rice in adults.

## Methods

### Participants

We used the CONSORT checklist when writing our article [14]. Participants were healthy adults (*n* = 26; 13 males and 13 females) aged 18–45 y with normal BMI (18.5–24.9 kg/m^2^). They were recruited through advertisements posted around the University of Toronto downtown campus and in Toronto Transit Commission trains. This study adhered to the guidelines in the Declaration of Helsinki and was registered as a clinical trial at ClinicalTrial.gov (ID: NCT05610124). All treatments and procedures were approved by the Human Participants Review Committee, Ethics Review Office, University of Toronto before commencement. The recruitment strategies and screening procedures were similar to those reported previously [9,15]. An in-person screening was conducted at the Department of Nutritional Sciences, University of Toronto, where written informed consent was obtained from all participants. Height (m) and weight (kg) were measured while participants were in light clothing and without shoes to determine BMI. Additionally, waist circumference (cm) and fasting BG (mmol/L) were measured as part of the baseline data collection.

Exclusion criteria included individuals who were irregular breakfast consumers, those actively attempting to gain or lose weight, elite athletes, smokers, individuals with fasting BG ≥5.5 mmol/L, and those with lactose intolerance or allergies to study foods. Additionally, individuals with irregular medication or protein supplementation routines, recent initiation of new medications or protein supplementation within the past 3 mo, any medication use or medical condition (such as diabetes) that could potentially influence study outcomes, current pregnancy or planning pregnancy, breastfeeding, and irregular menstrual cycles were excluded. Participants were also deemed ineligible if their reported restrained eating score exceeded 11 on the Eating Habits Questionnaire [16].

Mealtime FI and BG were the primary measures, whereas insulin, satiety, and FI at a later meal were the secondary measures. On the basis of our past experiments [17,18] in which participants’ mean FI was 900–1000 kcal at *ad libitum* meals, and power sample analysis for within-subject design, 26 subjects are required to detect a 150-kcal difference in FI between treatment meals (with a power of 80% and 2-sided significance level of 0.05). As FI following the treatment meals is a direct measure of satiety, it is important that our study is powered to detect a difference in this dependent measure between treatments. Additionally, on the basis of the results of previous experiments, this sample size (*n* = 26) is also sufficient to detect a 10% difference in subjective appetite ratings [measured by visual analog scales (VASs)] between treatments and control, with a power of 80% and an α of <0.05. Moreover, a sample size of 12 was determined to be sufficient to show a treatment effect of 10% on BG, the primary outcome measure, and on insulin with a power of 0.80 and an α of <0.05, on the basis of previous studies [[19], [20], [21]].

### Test meals

The test meals were prepared in the laboratory kitchen using commercial products according to the package instructions, as previously reported [9]. The ingredients of the commercial products used to prepare the treatment meals and pizza are listed in Supplemental Table 1. To prevent starch retrogradation, all test meals were served hot immediately after being removed from the oven, stove, or microwave. Each meal consisted of a fixed isocaloric portion of protein sources, accompanied by *ad libitum* access to 1 of the CHO sources.

Protein sources included: *1*) Lean beef meatballs, cooked in the oven for 12 min at 400^o^F using 124 g of PC President’s Choice Blue menu Angus beef meatballs (Loblaw Companies Limited). *2*) Lean beef meatballs cooked in the same manner using 114 g of PC President’s Choice Blue menu lean Italian beef meatballs (Loblaw Companies Limited) in the oven for 12 min at 400^o^F. This product replaced the Angus beef meatballs after their discontinuation from the market during the trial. *3*) Vegetable balls, cooked in the oven for 12 min at 204.4^o^C (400^o^F) using 133 g of IKEA—HUVUDROLL Vegetable Balls (IKEA). Participants were served a plate containing isocaloric amounts of either the animal or plant protein sources during each session and were instructed to consume the entire protein portion at an even pace over the 30-min mealtime. The selection of animal and plant protein sources was based on ensuring similar energy content. However, available plant-based substitutes with comparable palatability had lower protein content. Specifically, the animal protein source contained 26 g of protein per portion, whereas the plant protein source contained 10.4 g per portion. Despite the substantial difference in protein content, these amounts were chosen to provide similar energy content.

CHO sources included: *1*) BFF, prepared by baking 400 g of McCain Superfries Straight Cut French fries (McCain) in the oven for 18 min at 232.2^o^C (450^o^F) and then sprinkling evenly with 1/8 tsp of salt. *2*) IMP, prepared by boiling 187.5 g of water with 1/4 tsp of salt, and 15 g of unsalted butter (Gay Lea Foods Co-operative Ltd.). After removing from heat, 1/4 cup (53 g) of milk (3.25% TruTaste Homogenized Milk, Neilson, Saputo Dairy Products Canada G.P.) and 46 g of mashed potato dry mix (Betty Crocker—Dried mashed potato, General Mills) were added, followed by stirring for 2 min. *3*) Rice, prepared by microwaving 250 g of Uncle Ben’s—Ben’s Original Ready Rice white Basmati Rice (Mars, Inc.) for 1.5 min, then mixed with 10 g of unsalted butter (Gay Lea Foods Co-operative Ltd.), 1/8 tsp of salt, and 1/2 tsp dried vegetable seasoning (La Grille Seasoning, vegetable, Club house, McCormick Canada). Participants were served a total of 3 plates of 1 of the CHO sources during each session over a 30-min mealtime period (at 0 min, 10 min, and 20 min). Each plate contained 250 g of the CHO side, ready to eat, and was served with 300 g of water. Participants were instructed to eat as much of the CHO as they felt comfortably full and to drink as much water as they desired.

Two hours later, participants were served pizza meal *ad libitum*. The meal consisted of Dr. Oetker—Giuseppe Pizzeria Easy Pizzi Cheese or Pepperoni pizza (Dr. Oetker Canada Ltd.), prepared by baking in an oven at 232.2^o^C (450^o^F) for 8 min. Participants were served 3 plates of pizza over a 20-min mealtime period (at 120 min, 127 min, and 134 min). Each plate contained 2 pizzas of their preselected choice (cheese or pepperoni), with each rectangular pizza cut into 6 uniform squares and served in a random arrangement on a tray as previously reported [22]. Each plate of pizza was accompanied by 500 g of water. Participants were instructed to eat the pizza until comfortably full and to drink water *ad libitum*.

Energy and macronutrient composition of the meals and pizza as served were calculated on the basis of the manufacturer’s information from the Nutrition Facts table on the packaging and preparation instructions. The macronutrient composition of protein and CHO sources, as well as the pizza, are shown in Table 1. The weights of meatballs and their plant protein substitute (vegetable balls) were adjusted to provide similar a similar energy (kcal) content but were different in their protein contents.

**TABLE 1:** Energy and macronutrient composition^1^ of protein and carbohydrate sources in the treatment meals and of the pizza meal.

Characteristics	Beef meatball^2^^,^^3^	Vegetable ball^2^	Baked French fries	Instant mashed potatoes	Rice	Cheese pizza	Pepperoni pizza
Weight (g)	121	133	100	100	100	100	100
Energy (kcal)	240.0	240.0	152.9	99.1	188.5	235.3	240.8
Fat (g)	13	11.5	4.7	4.5	5.5	6.9	7.9
Carbohydrate (g)	6.6	23.0	25.9	12.8	31.9	32.6	31.9
Protein (g)	26.0	10.4	2.4	1.9	3.5	9.6	9.9
Fiber (g)	1.0	6.3	1.8	1.3	0.8	1.6	1.6

1 Macronutrient composition was calculated on the basis of information from the manufacturer and preparation instructions.

2 The weights of meatballs and their plant protein substitute (vegetable balls) were adjusted to provide similar amount of energy (kcal).

3 Mean composition of 2 meatballs products that were used.

### Experimental protocol

The study is a 2 × 3 factorial, single-blinded, randomized, controlled crossover acute trial, following a within-subject, repeated-measures design. True double blinding is not feasible in these study designs. Inherent differences in taste, smell, look and preparation of the various treatments are conspicuous and thus not possible to double-blind. All participants attended 6 sessions where the meals consisted of *ad libitum* servings of 1 of the 3 CHO sources: *1*) BFF, *2*) IMP, and *3*) rice (control), each served with a fixed isocaloric (240 kcal) amounts of either beef meatballs (animal protein) or vegetable balls (plant protein). The treatment order for the 6 sessions was randomly assigned for each participant by the study coordinator using a randomized block design generated with a random generator script in SAS version 9.4 (SAS Institute Inc.). Each treatment session was separated by a minimum of 1 wk to reduce order effects. The order of treatments was concealed from participants until they began eating at each session.

The study protocol and procedures were similar to those reported previously [23]. Participants attended the Department of Nutritional Sciences following a 12-h overnight fast, except for water, which was permitted until 1 h before each session. To minimize within-subject variability, all participants were scheduled to arrive at the same time and on the same day of the week for each treatment session. They were also instructed to maintain the same dietary and exercise patterns the evening before each session.

On 6 weekday mornings, each participant arrived at the laboratory between 0830 and 1030 h. As previously reported [24,25], participants completed baseline questionnaires upon arrival to ensure there were no unusual deviations from their diet and lifestyle patterns from the previous day and current morning. Participants who did not fast for 12 h or reported variances in sleep habits, stress level, normal activity, or diet the night before were rescheduled. Baseline questionnaires included “Sleep Habits and Stress Factors” and “Recent Food Intake and Activity Level,” as well as VAS assessing “physical comfort,” “energy, fatigue, and stress,” and “motivation to eat.” VAS for subjective appetite were administered at baseline (0 min) and at 30, 45, 60, 75, 90, 120, 140, 155, and 170 min. The “motivation to eat” VAS comprised 4 questions assessing desire to eat, hunger, fullness, and prospective food consumption and was used to calculate mean subjective appetite. The VAS used to assess appetite consisted of a 100-mm horizontal line with specific anchors at each end. Participants were instructed to mark a point on the line that best represented their current state for each question. The anchors for the appetite questions were as follows: for hunger—“Not hungry at all” (0 mm) to “As hungry as I have ever felt” (100 mm); for fullness—“Not full at all” (0 mm) to “Very full” (100 mm); for desire to eat—“Very weak” (0 mm) to “Very strong” (100 mm); for prospective food consumption—“Nothing at all” (0 mm) to “A large amount” (100 mm). Appetite scores were calculated on the basis of the mean of individual scales: subjective appetite = (hunger + (100 – fullness) + desire to eat + prospective food consumption)/4.

Participants were asked to remain seated for the duration of the study session and were permitted to engage in quiet activities, such as reading. Each participant provided a baseline finger-prick capillary blood sample to measure BG and ensure compliance to study procedures. A baseline measurement of >5.5 mmol/L indicated noncompliance with the fasting instructions, and the participant was asked to reschedule.

Following the finger-prick BG measurement, an indwelling intravenous catheter was inserted in the antecubital vein of 12 participants by a registered nurse to collect blood for later insulin analysis, and a baseline blood sample was obtained. Immediately thereafter, participants were escorted to a feeding room and seated in individual cubicles. Each participant was instructed to finish the protein source and to eat the CHO sides until feeling comfortably full.

Participants were first provided with a plate containing a 250 g serving of CHO sides (BFF, IMP, or rice) with either beef meatballs or vegetable balls and 300 g of water. Additional trays of freshly cooked CHO sides, each with an extra 300 g of water, were provided at 10 and 20 min, with the previous tray being removed. A palate cleanser (100 g of water) was provided with the last tray. Participants were instructed to drink water *ad libitum* but to finish the palate cleanser completely once they were done with their meal. They were given 30 min to complete the meal, simulating “at-home” meal consumption.

The pizza meal was served *ad libitum* 2 h later to determine the effects of treatment meals on FI, BG, and insulin levels during and after the second meal. Participants were given 20 min (from 120 to 140 min) to consume their pizza meal and were instructed to eat until they were comfortably full. A plate of pizza was served every 7 min, each accompanied by 500 g of water. Participants were instructed to drink water *ad libitum*. Each study visit lasted ∼3 h.

FI was assessed both during the meal and again during the *ad libitum* pizza consumption. The total amount of CHO sides and pizza consumed by each participant was determined by weighing and calculating the difference between the amount of food served and the leftovers during both the treatment meal and the pizza consumption. BG levels and subjective appetite were assessed at 30, 45, 60, 75, 90, and 120 min postmeal, and at 140, 155 and 170 min post-pizza, following the baseline measurements. Intravenous blood samples were collected at 0, 30, 60, 90, 120, 140, and 170 min for plasma insulin measurements.

### Blood biomarkers

Each subject provided a finger-prick capillary blood sample using a Mono-ejector Lancet device (Single-Let lancet; Ascensia Diabetes Care) at baseline (0 min) and at 30, 45, 60, 75, 90, 120, 140, 155, and 170 min. The plasma concentration of glucose was measured with a point-of-care glucose meter (Contour NEXT GEN Meter; Ascensia Diabetes Care). Glucometers were calibrated before each session to ensure accuracy. To maintain consistency across all study visits, each participant was assigned a specific glucometer and glucose strip lot number.

In a subsample of 12 participants, intravenous blood was collected by a registered nurse in lavender-capped BD evacuated tubes (BD Diagnostics), coated with EDTA at baseline (0 min) and at 30, 60, 90, 120, 140, and 170 min after the meals. The tubes were centrifuged at 2000 RCF (Micro high-speed refrigerated centrifuge, VS-15000CFNll, Vision Scientific Co., Ltd.) for 10 min at 4°C. Plasma samples were aliquoted to Eppendorf tubes and stored at −80°C for analysis. The plasma concentrations of insulin were measured using ELISA kits (intra-CV: 6.2%; inter-CV: 10.6%; no. 80-INSHU-E01.1, E10.1; ALPCO Insulin ELISA).

### Statistical analysis

Statistical analyses were conducted using SAS 9.4 (SAS Institute Inc.). Two-tailed paired *t* test was used to determine differences between sex groups for baseline participant characteristics. FI and palatability were analyzed using 2 and 3-factor analysis of variance (ANOVA) for the effects of protein, CHO, sex, and their interactions with a Tukey–Kramer’s *post hoc* analysis test. Subjective appetite scores were calculated on the basis of the mean of individual VAS using the equation: (hunger + (100 – fullness) + desire to eat + prospective food consumption)/4. Subjective appetite suppression per 100 kcal of treatment meal intake was calculated by subtracting subjective appetite scores from baseline values and then dividing the mean decrease in subjective appetite scores over 30–120 min by the respective calories of the treatment meal intake. [(baseline subjective appetite − mean subjective appetite) ÷ treatment calories in 100-kcal units]. The higher the value, the greater the appetite suppression.

For subjective appetite, subjective appetite suppression per 100 kcal, BG and plasma insulin data, 2-factor repeated measures analysis of covariance (ANCOVA) analysis was done for the means postmeal 30–120 min, and post-pizza 140–170 min to test for the effects of protein, CHO, time, and their interactions, followed by Tukey’s *post hoc* test. Moreover, 2-factor ANOVA analysis was done for the magnitude of change within the meal 0–30 min and within the pizza meal 120–140 min to test the effects of protein, CHO, and their interactions, followed by Tukey’s *post hoc* test. Sex was initially included as a factor affecting subjective appetite, palatability, BG, and plasma insulin; however, it was removed when there was no effect. Normality was determined using SAS PROC univariate Shapiro–Wilk test and when residuals were not normally distributed (*P* < 0.05), SAS PROC GLIMMIX for all ANCOVAs, and ANOVA. All results analyzed by ANCOVAs, and ANOVAs are presented as least-square means ± SEM. Statistical significance was concluded with a *P* value <0.05.

## Results

### Participants

Twenty-nine participants were recruited and 26 of them completed the study, including 13 males and 13 females, between March and November 2023. Dropouts included a total of 3 participants because of blood collection difficulties, loss to follow-up, and scheduling challenges. The study concluded after completing all study sessions for the intended sample size. FI, subjective appetite, and BG are presented for *n* = 26, and plasma insulin data are presented for a subset of *n* = 12 (6 males and 6 females) where intravenous blood collection was conducted. Baseline characteristics of participants in both parts of the study are shown in Supplemental Table 2.

Participant baseline characteristics (*n* = 26) are shown in Table 2. There were no differences (*P* > 0.05) between males (*n* = 13) and females (*n* = 13) in their age (y) and BG (mmol/L) at baseline. However, male participants had 5.6% higher height (cm), 15.8% higher body weight (kg), 5.5% higher BMI (kg/m^2^), and 12.6% higher waist circumference (cm) than female participants (*P* < 0.05).

**TABLE 2:** Participant characteristics at baseline^1^.

Characteristics	Females	Males	*P* value
Age (y)	23.62 ± 1.53	29.46 ± 2.29	0.0544
Height (cm)	164.1 ± 2.73	173.8 ± 1.15	0.0078
Weight (kg)	58.65 ± 2.39	69.63 ± 1.98	0.0033
BMI (kg/m^2^)	21.72 ± 0.50	22.99 ± 0.46	0.0303
Waist circumference (cm)	72.08 ± 1.55	82.49 ± 1.41	<0.0001
Blood glucose (mmol/L)	5.08 ± 0.07	5.17 ± 0.07	0.3571

1 Data are means ± SEM (*n* = 26). Significantly different (*P* < 0.05) between female and male participants, paired *t* test.

### FI

#### Meal intake

The meal energy intake was affected by CHO source (*P* < 0.0001), but not by protein source (*P* = 0.3108) or protein by CHO interaction (*P* = 0.3570). Energy intake from CHO was affected by CHO source (*P* < 0.0001), but not by protein source (*P* = 0.4469) or protein by CHO interaction (*P* = 0.3996) (Table 3). Participants consumed 23–25% more meal energy and 32–34% more CHO energy (*P* < 0.0001) at meals with rice compared with meals with BFF and mashed potatoes, which were similar.

**TABLE 3:** Effect of protein and carbohydrate source on meal, carbohydrate, and protein intake^1^.

	Carbohydrate source			*P* value		
	Baked French fries	Instant mashed potatoes	Rice	Protein	Carbohydrate	Protein by carbohydrate
Meal intake (kcal)	678.4 ± 29.74^a^	694.09 ± 27.43^a^	905.1 ± 48.47^b^	0.3108	<0.0001	0.3570
Carbohydrate intake (kcal)	436.2± 29.83^a^	451.9 ± 27.03^a^	666.9 ± 48.65^b^	0.4469	<0.0001	0.3996
Protein intake (kcal)	242.2 ± 1.90	242.1 ± 1.90	238.2 ± 1.90	0.0331	0.0737	0.3105

Abbreviation: ANOVA, analysis of variance.

Values in the same row with different superscript letters differ, *P* < 0.05.

1 Data are least-square means ± SEM, *n* = 26. A 2-factor ANOVA for meal, carbohydrate, and protein intake (kcal) was used with protein and carbohydrate as main factors. Values are means of meals with animal and plant protein sources.

#### Pizza intake

Pizza meal intake was not affected by protein source (*P* = 0.0781) or CHO source (*P* = 0.1563); however, there was a protein by CHO interaction (*P* = 0.0500) (Table 4). Although the animal protein-based meals resulted in similar energy intake at the pizza meal, the plant protein meals affected the energy intake at the pizza meal. Energy intake at the pizza meal after the treatment meal combination of plant protein and rice was 15.9% higher than that when plant protein was combined with BFF (*P* = 0.0303).

**TABLE 4:** Effect of protein and carbohydrate source on pizza and total energy intake (kcal)^1^, meal and pizza water intake (g)^2^.

	Carbohydrate source			*P* value		
	Baked French fries	Instant mashed potatoes	Rice	Protein	Carbohydrate	Protein × carbohydrate
Pizza intake (kcal)						
Animal protein	854.8 ± 55.20^a,b^	840.1 ± 55.20^a,b^	835.6 ± 55.20^a,b^	0.0781	0.1563	0.0500
Plant protein	814.4 ± 55.20^a^	902.4 ± 55.20^a,b^	968.6 ± 55.20^b^			
Total meal + pizza intake (kcal)^1^						
Animal protein	1530± 79.17	1534 ± 79.17	1763 ± 79.17	0.2897	<0.0001	0.3330
Plant protein	1495± 79.17	1596 ± 79.17	1850 ± 79.17			
Meal water intake (g)						
Animal protein	518.2 ± 49.35	469.6 ± 48.17	502.4 ± 52.82	0.8387	0.0204	0.4916
Plant protein	541.6 ± 45.79	457.5 ± 44.12	479.7 ± 51.40			
Pizza water intake (g)						
Animal protein	385.0 ± 49.46	400.7 ± 49.46	417.9 ± 49.46	0.6340	0.3567	0.1025
Plant protein	459.6 ± 49.46	344.9 ± 49.46	357.9 ± 49.46			

Values between the 2 protein and 3 carbohydrate types with different superscript letters differ, *P* < 0.05.

1 Total energy intake = energy intake of meal + pizza intake. Values are means of meals with animal and plant protein sources.

2 Data are least-square means ± SEM, *n* = 26. A 2-factor ANOVA for pizza and total energy intake (kcal), meal and pizza water intake (g) was used with protein and carbohydrate as main factors, and their interactions.

The combined energy intake of the meal and pizza was affected by the CHO source (*P* < 0.0001) but not by the protein source (*P* = 0.2897) or protein by CHO interaction (*P* = 0.3330) (Table 4). Meals with rice resulted in higher (*P* < 0.001) total energy intake compared with meals with either BFF or IMP. Meal combinations that included animal protein with BFF or IMP resulted in 233.41 kcal (13.23%) and 229.83 kcal (13.03%) less total energy intake, respectively, compared with meals with rice. Similarly, the meal combinations with plant protein resulted in 355.18 kcal (19.19%) and 253.95 kcal (13.72%) fewer total energy when consumed with BFF and IMP, respectively, compared with meals with rice (Table 4). Meal water intake was greater after BFF compared with IMP (*P* = 0.01189) and similar to rice (*P* = 0.2752). Pizza meal water intake was not affected by protein (*P* = 0.6340), CHO (*P* = 0.3567), or their interaction (*P* = 0.1025) (Table 4).

Energy intake from meal and CHO were affected by sex (*P* < 0.05), but not protein by sex or CHO by sex interactions (*P* > 0.05) (Table 5). Male participants consumed more meal energy (828.6 ± 42.65 kcal) than female participants (689.7 ± 42.65 kcal) (*P* = 0.0279), which is the result of more CHO energy (585.9 ± 42.97 kcal) than female participants (450.8 ± 42.97 kcal) (*P* = 0.034). Pizza meal intake was affected by sex (*P* = 0.0016), and a protein by sex interaction (*P* = 0.0248), but there was no CHO by sex interaction (*P* = 0.5431) (Table 5). Overall, male participants consumed more calories from pizza (1030.3 ± 64.17) than female participants (708.4 ± 64.17; *P* = 0.0016). The interaction revealed that male participants consumed higher pizza energy after meals with plant protein (1089.8 ± 67.18) than with animal protein in male (970.8 ± 67.18; *P* = 0.03) and female (716.2 ± 67.18; *P* = 0.0033) participants. On the other hand, female participants consumed lower pizza energy after meals with plant protein (700.5 ± 67.18) than male participants after animal protein (970.8 ± 67.18; *P* = 0.0415) and plant protein meals (1089 ± 67.18; *P* = 0.0022).

**TABLE 5:** Effect of sex, protein and carbohydrate source on meal, carbohydrate, protein, pizza, and total energy intake^1^.

	Carbohydrate source						*P* value		
	Baked French fries		Instant mashed potatoes		Rice		Sex	Sex × protein	Sex × carbohydrate
	Female	Male	Female	Male	Female	Male			
Meal intake (kcal)	599.7 ± 41.36	757.0 ± 41.36	637.7 ± 38.68	750.5 ± 38.68	831.9 ± 67.14	978.3 ± 67.14	0.0404	0.5809	0.7131
Carbohydrate intake (kcal)	360.9 ± 41.72	511.4 ± 41.72	394.4 ± 38.20	509.5 ± 38.20	596.9 ± 67.79	736.9 ± 67.79	0.0411	0.5975	0.8044
Protein intake (kcal)	240.1 ± 2.68	244.3 ± 2.68	244.8 ± 2.68	239.6 ± 2.68	237.3 ± 2.68	239.1 ± 2.68	0.9223	0.7961	0.0546
Pizza intake (kcal)	683.9 ± 70.06	985.3 ± 70.06	722.2 ± 70.06	1020 ± 70.06	719.1 ± 70.06	1085 ± 70.06	0.0016	0.0248	0.5431
Meal + pizza intake (kcal)^2^	1286 ± 103.4	1739± 103.4	1363± 103.4	1767± 103.4	1553± 103.4	2061 ± 103.4	0.0030	0.1003	0.4842

Abbreviation: ANOVA, analysis of variance.

1 Data are least-square means ± SEM, *n* = 26. A 3-factor ANOVA for pizza and total energy intake (kcal) was used with sex, protein, and carbohydrate as main factors, and their interactions.

2 Total energy intake = energy intake of meal + pizza. Values are means of meals with animal and plant protein sources.

The combined energy intake of the meal and pizza was affected by sex (*P* = 0.003) but there was no protein by sex interaction (*P* = 0.1003) or CHO by sex interaction (*P* = 0.4842) (Table 5). Male participants consumed more total calories from the meal and pizza than female participants (*P* = 0.003); male 1856 ± 97.26 compared with female 1401 ± 97.26.

#### Mean subjective appetite

**FIGURE 1:**
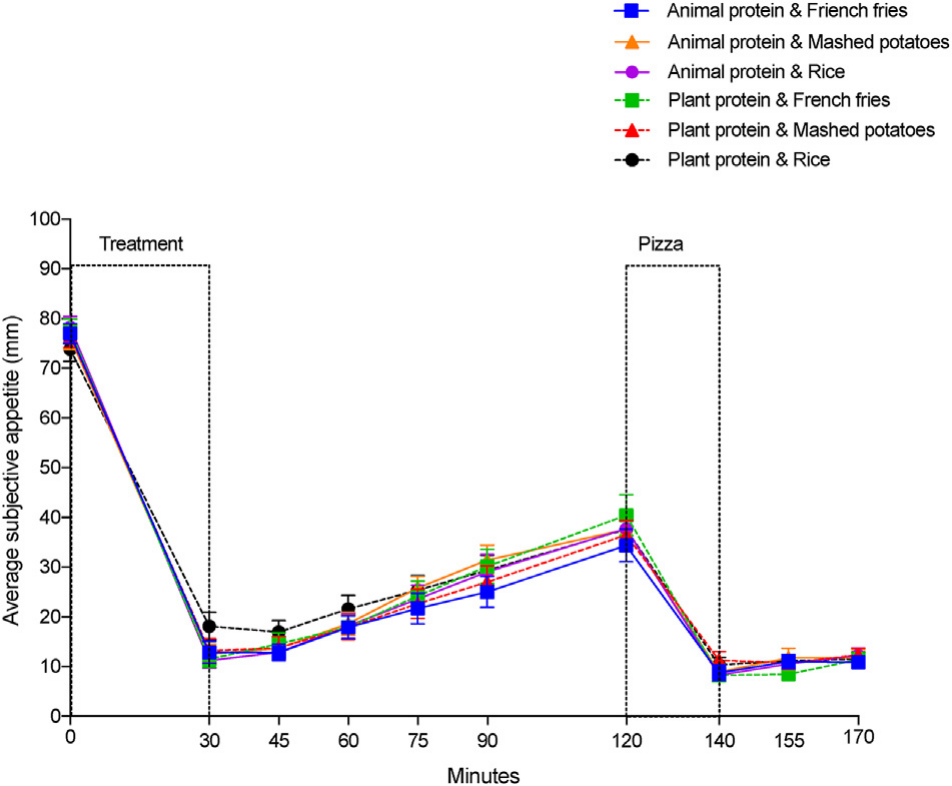
Mean subjective appetite over time. Values are means ± SEM; *n* = 26.

The mean subjective appetite (0–170 min) was affected by time (*P* < 0.0001), but not sex (*P* = 0.4544), protein source (*P* = 0.2237), CHO source (*P* = 0.2878), or any of their interactions (*P* > 0.05). Premeal appetite was highest at baseline, averaging 76.52 mm and decreased after the meal to 13.20 mm at 30 min and slowly rose to a mean of 37.33 mm at 120 min just before the pizza meal. Immediately after consuming the *ad libitum* pizza meal, the mean subjective appetite decreased to the lowest point averaging 9.25 mm at 140 min and slightly rose to a mean of 11.67 mm at the end of the study period (170 min) (Figure 1).

The mean subjective appetite magnitude of change (mm) within the meal 0–30 min was not affected by protein source (*P* = 0.1698) or CHO source (*P* = 0.4238), but there was a protein by CHO interaction (*P* = 0.0488). However, the Tukey–Kramer test did not show any significant differences between groups (Table 6). The subjective appetite means (mm) postmeal 30–120 min, the mean subjective appetite magnitude of change (mm) within the pizza meal 120–140 min, and the mean subjective appetite (mm) post-pizza meal (140–170 min) was not affected by protein source, CHO source, and no protein by CHO interaction (*P* > 0.05) (Table 6).

**TABLE 6:** Effect of protein and carbohydrate source on mean subjective appetite^1^.

	Carbohydrate source			*P* value		
	Baked French fries	Instant mashed potatoes	Rice	Protein	Carbohydrate	Protein × carbohydrate
Subjective appetite changes 0–30 min (mm)^2^	–65.08 ± 2.82	–63.52 ± 2.82	–61.39 ± 2.82	0.1698	0.4238	0.0488
Subjective appetite means 30–120 min (mm)^3^	22.04 ± 1.84	22.47 ± 1.84	23.34 ± 1.84	0.1562	0.3092	0.0566
Subjective appetite changes 120–140 min (mm)^2^	–28.91 ± 2.96	–26.93 ± 2.96	–28.39 ± 2.96	0.8584	0.6705	0.0627
Subjective appetite means 140–170 min (mm)^3^	9.76 ± 0.99	11.12 ± 0.99	10.60 ± 0.99	0.9893	0.0744	0.1290

Abbreviation: ANCOVA, analysis of covariance; ANOVA, analysis of variance.

^4^The Tukey–Kramer test did not show any differences between groups. Values are means of meals with animal and plant protein sources.

1 Data are least-square means ± SEM, *n* = 26.

2 Two-way ANOVA analysis for the subjective appetite magnitude of change within the meal 0–30 min and within the pizza meal 120–140 min to test the effects of protein, carbohydrates, and their interactions.

3 Two-way repeated measures ANCOVA analysis for subjective appetite means postmeal 30–120 min and post-pizza meal 140–170 min to test for the effects of protein, carbohydrates, time, and their interactions. The effect of time was significant (*P* = 0.0007); however; no time by protein, time by carbohydrates, or time by protein by carbohydrate interactions were found.

No main effects of sex were found at any time interval (Table 7). However, an interaction between sex and CHO source (*P* = 0.0462) in the postmeal (30–120 min) appetite scores was found. Nevertheless, the Tukey–Kramer test did not reveal any significant differences between groups. When data was separated into sex groups, there were no effect of protein (*P* = 0.2692), CHO (*P* = 0.2040), or their interaction (*P* = 0.2576) on the mean subjective appetite postmeal (30–120 min) among female participants. On the contrary, the mean subjective appetite postmeal (30–120 min) among males was not affected by protein source (*P* = 0.2601) or CHO source (*P* = 0.0784), but there was protein by CHO interaction (*P* = 0.0042). Male participants who consumed meals with plant protein and rice had higher mean subjective appetite scores during the postmeal (30–120 min) time interval than those who consumed meals with animal protein and rice (*P* = 0.0300).

**TABLE 7:** Effect of sex, protein, and carbohydrate source on mean subjective appetite^1^.

	Carbohydrate source						*P* value		
	Baked French fries		Instant mashed potatoes		Rice		Sex	Sex × protein	Sex × carbohydrate
	Female	Male	Female	Male	Female	Male			
Subjective appetite changes 0–30 min (mm)^2^	–64.36 ± 4.03	–65.79 ± 4.03	–60.13 ± 4.03	–66.92 ± 4.03	–60.10 ± 4.03	–62.68 ± 4.03	0.4487	0.7438	0.6139
Subjective appetite means 30–120 min (mm)^3^	19.28 ± 2.59	24.79 ± 2.59	21.74 ± 2.59	23.20 ± 2.59	20.83 ± 2.59	25.87 ± 2.59	0.2679	0.7048	0.0462
Subjective appetite changes 120–140 min (mm)^2^	–25.25 ± 3.98	–32.57 ± 3.98	–22.06 ± 3.98	–31.80 ± 3.98	–23.10 ± 3.98	–33.68 ± 3.98	0.0705	0.8532	0.7470
Subjective appetite means 140–170 min (mm)^3^	10.30 ± 1.38	9.21 ± 1.38	12.77 ± 1.38	9.47 ± 1.38	12.27 ± 1.38	8.94 ± 1.38	0.1734	0.9168	0.1038

Abbreviation: ANCOVA, analysis of covariance; ANOVA, analysis of variance.

^4^The Tukey–Kramer test did not show any differences between groups. Values are means of meals with animal and plant protein sources.

1 Data are least-square means ± SEM, *n* = 26.

2 Three-way ANOVA analysis for the subjective appetite magnitude of change within the meal 0–30 min and within the pizza meal 120–140 min to test the effects of sex, protein, carbohydrates, and their interactions.

3 Three-way repeated measures ANCOVA analysis for subjective appetite means postmeal 30–120 min and post-pizza meal 140–170 min to test for the effects of sex, protein, carbohydrates, time, and their interactions. The effect of time was significant (*P* = 0.0007); however, no time by protein, time by carbohydrates, or time by protein by carbohydrate interactions were found.

Two-way repeated measures ANCOVA analysis for subjective appetite suppression means per 100 kcal of treatment meal intake post-treatment meal 30–120 min was done to test for the effects of protein, CHO, time, and their interactions. The effect of time was significant (*P* = 0.0305); however; no time by protein, time by CHO, or time by protein by CHO interactions were found.

The subjective appetite suppression per 100 kcal was not affected by the CHO source (*P* = 0.1781) but was affected by the protein source (*P* = 0.0405) and by a protein by CHO interaction (*P* = 0.0274). The subjective appetite suppression per 100 kcal after meals with animal protein (–7.956 ± 0.2301) was significantly higher than the suppression after meals with plant protein (–7.693 ± 0.2301, *P* = 0.0405). The subjective appetite suppression per 100 kcal after meals with animal protein and rice (–8.236 ± 0.2626) was significantly higher than that after both meals with animal protein and mashed potatoes (–7.570 ± 0.2608) (*P* = 0.0373) and meals with plant protein and rice (–7.590 ± 0.2652) (*P* = 0.0402) (data not shown here).

#### BG

As sex was not a factor at any time interval (*P* > 0.05), BG data was pooled to test the effects of protein, CHO, time, and their interactions. BG concentrations (mmol/L) over time (0–170 min) were affected by time (*P* < 0.0001), protein source (*P* = 0.0139), CHO source (*P* < 0.0001), and time by CHO interaction (*P* = 0.0348). However, no protein by CHO (*P* = 0.1544) and no time by protein interactions (*P* = 0.2970) were found.

**FIGURE 2:**
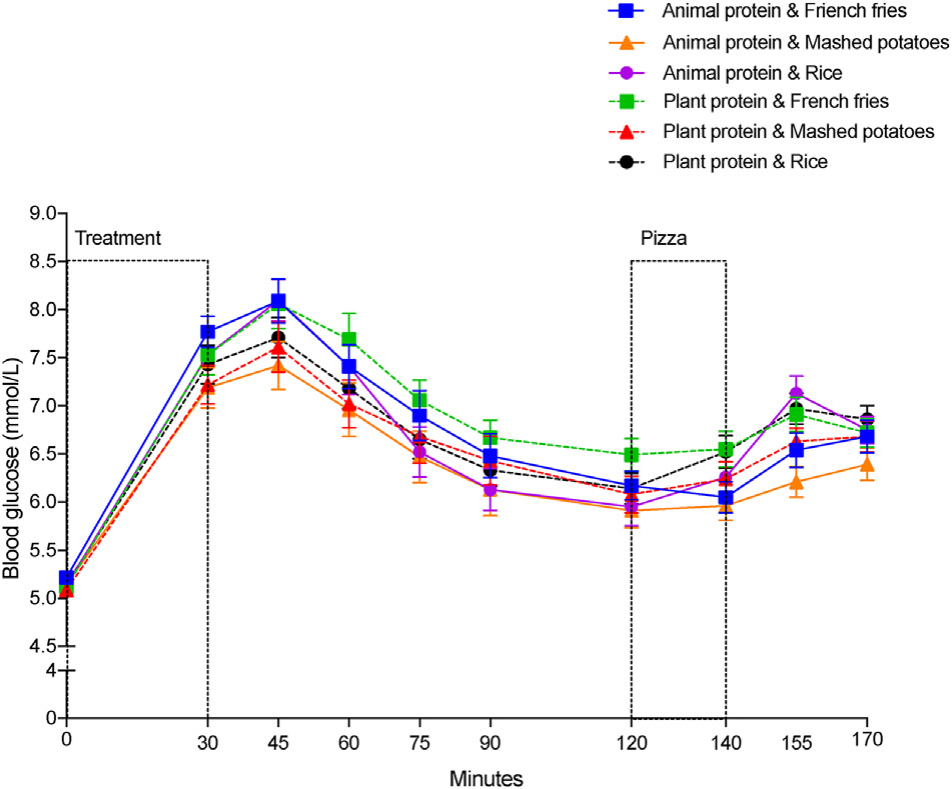
Mean blood glucose levels over time. Values are means ± SEM; *n* = 26.

BG peaked at 45 min after all treatment meals (Figure 2). The magnitude of change in BG within the meal 0–30 min was affected by CHO source (*P* = 0.0334), but not by protein source (*P* = 0.6833) or a protein by CHO interaction (*P* = 0.7051) (Table 8). BG increase was higher after BFF (2.48 ± 0.17 mmol/L) compared with IMP (2.08 ± 0.17 mmol/L) (*P* = 0.0271), but both were similar to rice (2.33 ± 0.17 mmol/L) (*P* > 0.05).

**TABLE 8:** Effect of meals on blood glucose^1^.

	Carbohydrate source			*P* value		
	Baked French fries	Instant mashed potatoes	Rice	Protein	Carbohydrate	Protein × carbohydrate
Blood glucose changes 0–30 min (mmol/L)^2^	2.48 ± 0.17^b^	2.08 ± 0.17^a^	2.33 ± 0.17^a,b^	0.6833	0.0334	0.7051
Blood glucose means 30–120 min (mmol/L)^3^	7.19 ± 0.15^b^	6.76 ± 0.15^a^	6.92 ± 0.15^a^	0.1559	<0.0001	0.3615
Blood glucose changes 120–140 min (mmol/L)^2^	–0.03 ± 0.12^a^	0.10 ± 0.12^a,b^	0.34 ± 0.12^b^	0.3280	0.0487	0.9255
Blood glucose means 140–170 min (mmol/L)^3^	6.51 ± 0.09^a^	6.39 ± 0.09^a^	6.77 ± 0.09^b^	0.0076	<0.0001	0.1459

Abbreviation: ANCOVA, analysis of covariance; ANOVA, analysis of variance.

Values in the same row with different superscript letters differ, *P* < 0.05. Values are means of meals with animal and plant protein sources.

1 Data are least-square means ± SEM, *n* = 26.

2 Two-way ANOVA analysis for the blood glucose magnitude of change within the meal 0–30 min and within the pizza meal 120–140 min to test the effects of protein, carbohydrates, and their interactions.

3 Two-way repeated measures ANCOVA analysis for blood glucose means postmeal 30–120 min and post-pizza meal 140–170 min to test for the effects of protein, carbohydrates, time, and their interactions. The effect of time was significant (*P* < 0.0001); however, no time by protein, time by carbohydrates, or time by protein by carbohydrate interactions were found.

BG mean concentrations postmeal (30–120 min) were affected by CHO source (*P* < 0.0001), but not protein (*P* = 0.1559) or a protein by CHO interaction (*P* = 0.3615) (Table 8). Post-treatment BG was higher after meals with BFF (7.19 ± 0.15 mmol/L) compared with meals with IMP (6.76 ± 0.15 mmol/L) (*P* < 0.0001) and rice (6.92 ± 0.15 mmol/L) (*P* = 0.0012), which were similar. Moreover, the BG change within the pizza meal (120–140 min) was affected by the CHO source (*P* = 0.0487), but not by the protein source (*P* = 0.3280) or a CHO by protein interaction (*P* = 0.3280) (Table 8). The BG change during the pizza meal was significantly lower after BFF (–0.03 ± 0.12 mmol/L) than that after rice (0.34 ± 0.12 mmol/L) (*P* = 0.0402). Meals with BFF led to the lowest BG change within the pizza meal (120–140 min), followed by meals with IMP, and then meals with rice.

BG means post-pizza meal (140–170 min) were affected by both the protein source (*P* = 0.0076) and the CHO source (*P* < 0.0001), but no protein by CHO interaction (*P* = 0.1459). Meals with animal protein led to lower BG means (6.48 ± 0.09 mmol/L) post-pizza meal (140–170 min) than meals with plant protein (6.64 ± 0.09) (*P* = 0.0076). Post-pizza BG was the highest after meals with rice (6.77 ± 0.09 mmol/L) compared with both meals with BFF (6.51 ± 0.09 mmol/L) (*P* = 0.0012) and meals with IMP (6.39 ± 0.09 mmol/L) (*P* < 0.0001).

#### Plasma insulin

**FIGURE 3:**
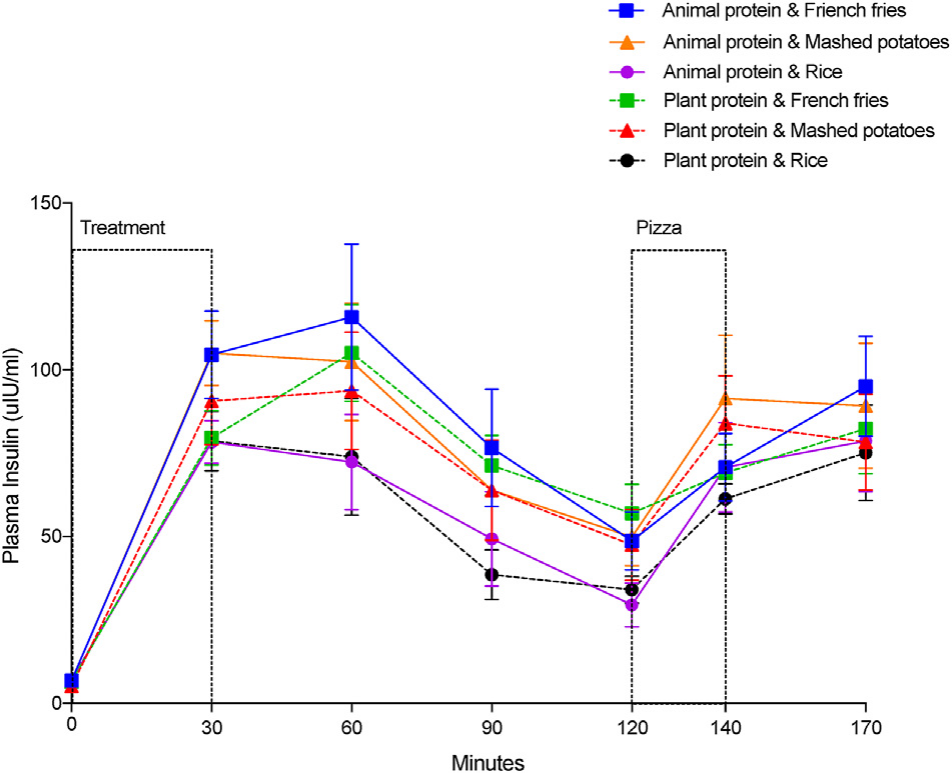
Mean plasma insulin levels over time. Values are means ± SEM; *n* = 12.

The plasma insulin concentrations (μIU/mL) over time are shown in Figure 3. Plasma insulin peaked at 30–60 min (Figure 3). Over time (0–170 min), it was affected by time (*P* < 0.0001), CHO source (*P* = 0.003), a time by CHO interaction (*P* = 0.016), and a sex by protein interaction (*P* = 0.0376), but not by protein source (*P* = 0.0797), or a protein by CHO interaction (*P* = 0.7820), or sex (*P* = 0.9948).

The plasma insulin increases within the meal 0–30 min was affected by the CHO source (*P* = 0.0420), but not by the protein source (*P* = 0.0730), or a protein by CHO interaction (*P* = 0.2866) (Table 9). The increase after the treatment meal was higher after the 2 meals with IMP (91.87 ± 8.34 μIU/mL) compared with the 2 meals with rice (71.59 ± 8.34 μIU/mL) (*P* = 0.0371), but both were similar to meals with BFF (85.39 ± 8.34 μIU/mL) (*P* > 0.05).

**TABLE 9:** Effect of meals on plasma insulin^1^.

	Carbohydrate source			*P* value		
	Baked French fries	Mashed potatoes	Rice	Protein	Carbohydrate	Protein × carbohydrate
Plasma insulin changes 0–30 min (μIU/mL)^2^	85.39 ± 8.34^a,b^	91.87 ± 8.34^b^	71.59 ± 8.34^a^	0.0730	0.0420	0.2866
Plasma insulin means 30–120 min (μIU/mL)^3^	82.16 ± 8.58^b^	77.75 ± 8.60^b^	56.44 ± 8.59^a^	0.2992	<0.0001	0.8035
Plasma insulin changes 120–140 min (μIU/mL)^2^	17.14 ± 6.90^a^	39.03 ± 6.90^b^	34.21 ± 6.90^b^	0.0875	0.0056	0.7839
Plasma insulin means 140–170 min (μIU/mL)^3^	70.39 ± 6.43	81.23 ± 6.37	84.98 ± 6.53	0.0406	0.0794	0.7434

Abbreviation: ANCOVA, analysis of covariance; ANOVA, analysis of variance.

Values in the same row with different superscript letters differ, *P* < 0.05. Values are means of meals with animal and plant protein sources.

1 Data are least-square means ± SEM, *n* = 26.

2 Two-way ANOVA analysis for the plasma insulin magnitude of change within the meal 0–30 min and within the pizza meal 120–140 min to test the effects of protein, carbohydrates, and their interactions.

3 Two-way repeated measures ANCOVA analysis for plasma insulin means postmeal 30–120 min and post-pizza meal 140–170 min to test for the effects of protein, carbohydrates, time, and their interactions. The effect of time was significant (*P* < 0.0001); however, no time by protein, time by carbohydrates, or time by protein by carbohydrate interactions were found.

Postmeal plasma insulin means (30–120 min) were not affected by protein (*P* = 0.2992) and no protein by CHO interaction (*P* = 0.8035) was found (Table 9). However, they were affected by the CHO source (*P* < 0.0001). Postmeal plasma insulin was the highest after meals with BFF (82.16 ± 8.58 μIU/mL) and IMP (77.75 ± 8.60 μIU/mL) compared with meals with rice (56.44 ± 8.59 μIU/mL) (*P* < 0.002).

Plasma insulin change within the pizza meal (120–140 min) was not affected by the protein source (*P* = 0.0875) or a protein by CHO interaction (*P* = 0.7839) but was affected by the CHO source (*P* = 0.0056) (Table 9). Meals with BFF led to the lowest change in plasma insulin (17.14 ± 6.90 μIU/mL) within the pizza meal (120–140 min), compared with meals with IMP (39.03 ± 6.9 μIU/mL) (*P* = 0.0060) and rice (34.20 ± 6.90 μIU/mL) (*P* = 0.0336), which were similar.

Post-pizza (140–170 min) plasma insulin means were not affected by treatment meal CHO source (*P* = 0.0794) or CHO by protein interaction (*P* = 0.7434). However, they were affected by the protein source (*P* = 0.0406) (Table 9). Meals with animal protein led to higher mean plasma insulin post-pizza meal (140–170 min) than meals with plant protein (84.54 ± 5.87 compared with 73.18 ± 5.87 μIU/mL).

## Discussion

The results of this study underscore the significance of meal composition, particularly the CHO source, in influencing PPG, satiety, and short-term FI. They confirm our hypothesis that PPG response during and after an *ad libitum* meal containing either potatoes or rice are not predictable on the basis of their GI when consumed alone. Meals with potatoes (BFF and IMP), irrespective of the co-ingested protein source, resulted in lower meal energy and CHO energy intake compared with meals with rice. This might be attributed in part to the higher fiber content of the BFF (1.8 g) and IMP (1.3 g) compared with rice (0.8 g) as prepared per 100 g. This aligns with our findings in children [9], where boiled mashed potato with meat led to a 40% reduction in meal energy intake compared with meat and rice. It also corresponds to a study in adult men, demonstrating that energy consumed at an *ad libitum* potato meal with 150 g of meat was 31% and 23% lower than pasta and rice meals, respectively [11].

Total energy intake at the meal and pizza mirrored the pattern of meal energy intake. Meals with BFF or IMP resulted in 294.3 kcal (16.3%) and 241.9 kcal (13.4%) less total energy intake, respectively, compared with meals with rice. This might be attributed, in part, to the higher energy content of the rice side dish compared with both BFF and IMP as prepared per 100 g. However, the reduced energy intake within the meals with potatoes was not compensated for at the pizza meal. These results align with a study in children and adolescents, where the total daily energy intake was 291 kcal (11%) lower after meals with potatoes and eggs compared with a control meal [12]. The results are also in agreement with a study conducted on men, which showed that FI at a boiled potato meal with meat was 40% lower compared with rice and pasta with meat [11].

Postprandial glucose and insulin concentrations are primarily determined by the amount and availability of CHO in foods [26]. However, this does not predict meal outcomes in an *ad libitum* environment where meals contain protein, fiber, and other components of food. BG increase at the meal, during the postmeal interval and the pizza meal was the highest after BFF followed by rice and the lowest after IMP. Insulin was higher in these same intervals after IMP and BFF than rice. Post the pizza meal, both BG and insulin were the highest following the rice meal compared with both meals with IMP and BFF.

It is unsurprising that the IMP meal resulted in the lowest BG levels both within and after the meal, given its lower energy intake at the meal, resulting in lower CHO consumption. Furthermore, the IMP, as prepared, provided a lower CHO load (12.8g/100 g) compared with BFF (25.9 g CHO/100 g) and rice (31.9 g CHO/100 g) at the meal. Although BG response was similar after BFF and rice, insulin levels were higher after BFF than after rice, potentially contributing to the lower FI during the meal. However, considering that BG is a well-known satiety signal, it is noteworthy that BG was the lowest after the rice meal, which may explain the observed 34% higher CHO during meals with rice compared with meals with BFF.

Following the treatment meal, BFF when compared with IMP, resulted in sustained higher mean BG and insulin over time. This contrasts with that expected as the BFF contained 25% of energy from fat, had higher fiber content, and were baked from frozen. These factors are known to lower the glycemic response to potatoes. Pre-frying and freezing increase the amount of resistant starch in frozen French fries, and amylose reacts with lipids to form amylose-lipid complexes [7,27] that are digested at a slower rate [28,29]. However, the result may be attributed to the baking process using dry heat [30], and causing loss of water, which, in turn, concentrates free sugars [28]. Moreover, starch gelatinization through cooking and processing increases the susceptibility of starch to digestive enzymes, making the BFF more readily digested by amylase enzymes compared with uncooked starch [31]. Furthermore, the results may not reflect the responses to fries cut from potatoes at home and deep fried for immediate consumption. Our previous study in children found that BFF, compared with fried French fries, also resulted in higher BG and insulin responses than after pasta [9].

The lasting effect of a meal on later meal metabolic response is illustrated by these results, suggesting that this is a factor to consider in designing meal patterns for glycemic control. Although BFF resulted in the highest BG and insulin before the pizza meal, it resulted in a lower change in BG and insulin than meals with rice within and following the later pizza meal. This high insulin level might explain the lower and sustained post-pizza BG after meals with BFF and meals with IMP compared with meals with rice. Although limited studies have investigated the glycemic response to meals with potatoes after the second meal, the association between glycemic response to the first meal and subsequent FI and glycemic control is more clearly seen when CHO are consumed alone rather than in a complex meal [20]. Furthermore, although the effect of protein and fat consumption as part of the test meals on the glycemic response has been reported earlier [32], only French fries served with a fixed portion of egg omelet led to lower total daily energy intake compared with a control breakfast meal with cereal, milk, and bread in a previous study [12]. Further research investigating the glycemic and appetite responses to meals with potatoes after the second meal for longer time intervals might be beneficial. However, BG tended to be lower after the boiled potato meal and postprandial insulin was markedly reduced, reflecting their reduced CHO intake [11].

Meals with animal protein had a lasting effect at the second meal, resulting in lower BG and higher plasma insulin levels post-pizza meal (140–170 min) than meals with plant protein. However, this can be explained by the lower CHO and higher protein content in the beef meatballs (26 g/portion) compared with the vegetable balls (10.4 g/portion). It is possible that the glucose-dependent insulinotropic polypeptide and glucagon-like peptide-1 may have influenced these responses [33]; however, they were not measured in this study. Moreover, it remains to be determined if a plant-based substitute with the equivalent protein content and equivalent palatability to the animal-based protein would result in similar PPG.

Moreover, it is well-documented that the palatability of food is another significant determinant of energy intake [34]. When the pleasantness ratings of the CHO sources were tested, meals with rice had a higher rating compared with both BFF and IMP in the current study (data not shown), which might have contributed to the higher rice energy intake. On the other hand, when the palatability of the protein sources was tested, both sources had similar ratings. However, the ratings of the CHO sources taste and texture were significantly higher when combined with animal protein compared with plant protein, which might have contributed to the higher CHO and meal energy intake at meals with animal proteins compared with meals with plant proteins, although statistically nonsignificant.

Subjective appetite scores failed to show an effect of the treatment, even though the number of participants were sufficient to detect 10% differences among treatments. This may be explained by their *ad libitum* eating leading to maximized satiation. Nevertheless, the large difference in energy intake at the rice meal compared with the BFF and IMP meals can be attributed to physiologic response not captured by the subjective appetite scores. In a study assessing the impact of 4 isocaloric potato-based meals (fried French fries, baked potato, mashed potato, or potato wedges) on subjective satiety sensations and subsequent energy intake at an *ad libitum* meal, the results indicated that the meal containing fried French fries was perceived to be significantly more satiating compared with the isocaloric pasta-based control meal. The other potato-based meals did not show a significant difference in satiety compared with the control meal. All test meals had a comparable effect on energy intake at a later *ad libitum* meal [10]. A literature review concluded that isoenergetic portions of potatoes, in particular boiled potatoes, are more satiating than other starchy CHO when consumed in isolation. Similarly, when *ad libitum* consumption was permitted, as in the present study, less energy was consumed in mixed meals incorporating potato, without a compensatory increase in energy intake at a subsequent meal, despite lower satiety ratings [2].

Although treatment meals showed no significant effects on the mean subjective appetite, meals with potatoes (BFF and IMP) resulted in lower meal energy and CHO energy intake compared with meals with rice. Conversely, when potatoes were consumed as part of an *ad libitum* mixed meal, they increased satiety per kcal and reduced FI compared with other CHO foods (pasta or rice) in adults [10,11] and children [9,12]. These effects may be attributed to the interaction of potato components. For instance, potato protease inhibitor II has been shown to increase satiety [35] and decrease FI [36], likely because of the delayed gastric emptying and increased circulating levels of the satiety hormone, cholecystokinin (CCK) [37]. However, in our previous study with children, although boiled mashed potatoes co-ingested with beef increased satiety compared with all other CHO sources (rice, pasta, BFF, and fried French fries), neither ghrelin nor peptide YY levels predicted the effect on FI suppression [9].

On the other hand, because of the different energy densities of the CHO sources, the weight of the IMP intake (g) exceeded that of other CHO sources (g) in both animal and plant protein meals (*P* < 0.0001) (data not shown here). The role of energy density and volume as determinants of FI did not align with the volumetric hypothesis of meal intake regulation. Numerous studies have demonstrated that adding water within a freely eaten meal reduces energy intake without affecting the amount of food consumed [38,39]. Corresponding with this hypothesis, the highest weight-to-energy intake ratio was observed in the IMP meals. This finding is consistent with our previous study in children, where the boiled mashed potato meal resulted in the highest weight and lowest energy intake compared with all other CHO that included rice, pasta, BFF, and fried French fries [9]. It is noteworthy that the greatest weight of food was consumed at the IMP meal. However, because of the higher energy density of rice, the rice meal resulted in a higher caloric intake. This finding suggests that the volume of food may not be the primary factor regulating intake. Instead, it underscores the potential importance of consuming foods with lower energy densities, which can be consumed in larger volumes while providing fewer calories.

Furthermore, the protein content of the treatment meals may have affected satiety and FI suppression. High-protein meals are known to induce greater satiety compared with low-protein meals, with some studies suggesting that protein has a maximal effect on increasing satiety and lowering FI when intakes range between 30 and 49 g [40]. In the present study, the meals contained a total of 32–36 g of protein from both the protein and CHO components. The energy contribution from the protein, expressed as a percentage of total meal calories, was 35% and 26% following the potato and rice meals, respectively. This indicates that the protein source contribution to the total meal energy was higher in meals with potatoes compared with meals with rice. Despite this, the mean subjective appetite suppression per 100 kcal of treatment meal was not affected by the CHO source but was significantly affected by the protein source. The subjective appetite suppression per 100 kcal after meals with animal protein was higher than after meals with plant protein. This difference can be explained on the basis of the variation in protein content between the isocaloric protein sources, with animal protein providing 26 g of protein per portion and plant protein providing 10.4 g per portion. The effect of CHO and protein sources on FI regulation may be mediated through the release of gastrointestinal hormones, which may increase anorexigenic hormones and/or decrease orexigenic hormones [41].

Although the fat content of the CHO sources was similar, the fat source may have affected total energy intake of the meal and pizza; only BFF were prepared in canola oil, which is high in monounsaturated and polyunsaturated fatty acids, compared with IMP and rice that were prepared with butter (made up of saturated fatty acids). Polyunsaturated fatty acids lower FI in adults compared with monounsaturated and saturated fatty acids [42], in part, through the release of CCK [43]. This might explain our finding that BFF resulted in the lowest meal energy and the lowest CHO energy intake followed by IMP and rice. Future studies measuring other gastrointestinal hormones may help contribute to an understanding of the physiologic factors affecting FI control in this study.

Furthermore, the consumption of fat with CHO foods delays starch degradation by slowing gastric emptying [44] and reduces postprandial glucose with no effect on the insulin response [45]. Because rice provided a slightly higher amount of fat (5.5 g/100 g rice) at the meal compared with other CHO sides (BFF: 4.7g/100 g; IMP: 4.5 g/100 g), and participants consumed more rice than other CHO sides, the delayed gastric emptying after rice meals might explain the similarity between the postmeal BG levels after rice meals and after IMP meals despite the lower CHO content of IMP. Moreover, meals with rice resulted in the lowest increase in plasma insulin within and after treatment meals. These findings are in line with a study in healthy male volunteers who consumed combinations of main dishes containing a moderate amount of fat and vegetable dishes with boiled white rice to investigate their effects on postprandial plasma glucose, insulin, and incretin hormone responses. Researchers found these meal combinations to be beneficial for lowering postprandial glucose concentrations, without excessive increase in insulin response [45].

The strength of this study design lies in its utilization of marketplace products that a consumer might use to prepare at-home meals. Although the present study examined the effects of different CHO side dishes in the context of mixed meals, there are some limitations. First, although the protein sources of the treatment meals were matched for energy, the CHO side dishes were served *ad libitum*, and they were not matched for volume or energy density. Previous studies suggest that both water content [8] and energy density [34,46] are determinants of FI regulation. IMP have high water content resulting in a larger volume and lower energy density, which may explain their effect on short-term satiety and energy intake. Second, we did not examine the effects of these treatment meals in obese or overweight adults, who may respond dissimilarly to the effects of meal composition compared with adults in the healthy weight range [47]. Lastly, this was a short-term study examining the effects of a single morning meal on FI, satiety, and BG and insulin levels over almost 3 h in a controlled laboratory setting. The long-term effects of the regular consumption of potato products on satiety, FI, glycated hemoglobin, and body weight and composition need to be studied.

This research challenges prevailing dietary recommendations, offering a nuanced understanding of the role of potatoes in overall meal consumption and its subsequent impact on metabolic and satiety markers. The findings call for a holistic approach to evaluating the effects of meals on short-term and later metabolic responses and FI. These insights have the potential to reshape dietary guidelines and promote a more accurate perspective on the health implications of potato consumption within the broader context of meal composition and their benefits in vegetarian and plant-based diets. Dietary guidelines should not discourage potato consumption but rather highlight the benefits of including potatoes as part of a balanced diet. Although pasta and rice also have important roles in the diet, especially from cultural perspectives and because of their contributions of resistant starch and fiber, potatoes provide a higher nutrient content relative to their calorie content. Encouraging the consumption of potatoes alongside other nutrient-rich foods, such as vegetables, can enhance the overall dietary quality.

In conclusion, adults consuming meatballs or plant-based substitutes with *ad libitum* IMP had lower PPG post-treatment and at a later pizza meal than when consumed with rice. Both IMP and BFF resulted in lower energy intake than after rice. These findings suggest that the inclusion of potato-based dishes with protein may play a significant role in regulating energy intake and postprandial BG response. Further research is needed to explore the underlying mechanisms and potential implications for dietary guidelines.

## Data Availability

Data described in the manuscript, code book, and analytic code will be made available upon request pending (for example, application and approval, payment, and others).
