# Supplementary material for: Potatoes Compared with Rice in Meals with either Animal or Plant Protein Reduce Postprandial Glycemia and Increase Satiety in Healthy Adults: A Randomized Crossover Study
Source: J Nutr. 2024 Aug 23;154(10):2999–3011. doi: 10.1016/j.tjnut.2024.08.017 (PMC11522892; doi:10.1016/j.tjnut.2024.08.017)
Supplement: multimedia component 1 [file mmc1.docx]

**Supplementary Materials**

**Supplemental Table 1.** List of ingredients of commercial products used to prepare treatment and pizza meals.

| **Commercial product name** | **Product description** | **Ingredients list** |
| --- | --- | --- |
| PC® - Blue Menu Angus Beef Meatballs | Beef meatballs | Angus beef, water, soy flour, brown rice crumbs, flavour, salt, soy protein, spice, spice extract, soy lecithin. |
| PC® - Blue Menu Italian Beef Meatballs | Beef meatballs | Beef, water, textured soy flour, toasted wheat crumbs, seasoning (parmesan cheese (milk), spices, dehydrated garlic and onion, parsley), soy protein concentrate, salt. |
| IKEA® - HUVUDROLL  Vegetable balls | Vegetable balls | Chickpeas, raw carrots, garden peas, sweet peppers, sweet maize, kale, pea protein, yellow onion, rapeseed oil, pea starch, salt, methyl cellulose, brown sugar from beets, yeast extract, onion powder, dried sage, black pepper, dried carrot, dried bay leaves, dried parsley, turmeric root. |
| McCain® - Superfries Straight Cut French fries | French fries | Potatoes, Canola oil, Sea salt, Sodium phosphate |
| Betty Crocker® - Dried mashed potato | Instant mashed potatoes | Dried potatoes, mono and diglycerides, sodium phosphate, citric acid |
| Uncle Ben’s® - Ben’s Original™ Ready Rice™ Basmati Rice | Instant rice | Water, Basmati rice; less than 2% of: Canola oil and/or sunflower oil, soy lecithin. |
| Dr. Oetker - Giuseppe Pizzeria Easy Pizzi Cheese | Cheese pizza | Enriched wheat flour (barley), Water, Part skim mozzarella cheese (partly skimmed milk, bacterial culture, salt, microbial enzyme), Tomato paste, Cheddar cheese (milk, bacterial culture, salt, microbial enzyme, calcium chloride, annatto), Sugar, Monterey Jack cheese (milk, bacterial culture, salt, microbial enzyme), Soya oil, Salt, Yeast, Corn starch, Spices and herbs, Garlic powder, Natural flavour. Contains: Wheat, Barley, Milk. |
| Dr. Oetker - Giuseppe Pizzeria Easy Pizzi Pepperoni | Pepperoni pizza | Enriched wheat flour (barley), Water, Part skim mozzarella cheese (partly skimmed milk, bacterial culture, salt, microbial enzyme), Pepperoni, Tomato paste, Sugar, Soya oil, Salt, Yeast, Corn starch, Spices and herbs, Garlic powder, Natural flavour. Contains: Wheat, Barley, Milk. |

**Supplemental Table 2.** Participant characteristics according to sex and method of blood collection.

|  | **Non-Intravenous blood collection (n = 14)** | | **Intravenous blood collection (n = 12)** | |
| --- | --- | --- | --- | --- |
| **Characteristics^1^** | **Females** | **Males** | **Females** | **Males** |
| N | 7 | 7 | 6 | 6 |
| Age, y | 21.57 ± 1.15 | 28.43 ± 3.21 | 26.00 ± 2.88 | 30.67 ± 3.50 |
| Height, cm | 161.61 ± 3.60 | 172.27 ± 0.90 | 166.92 ± 4.20 | 175.75 ± 2.10 |
| Weight, kg | 54.57 ± 2.54 | 68.51 ± 2.37 | 63.42 ± 3.50 | 70.93 ± 3.44 |
| BMI, kg/m^2^ | 20.89 ± 0.70 | 23.07 ± 0.69 | 22.69 ± 0.53 | 22.89 ± 0.66 |
| Waist circumference, cm  Blood glucose, mmol/l | 68.53 ± 1.42  5.03 ± 0.08 | 82.13 ± 1.30  5.14 ± 0.08 | 76.22 ± 1.84 | 82.92 ± 2.82 |
|  |  |  | 5.13 ± 0.12 | 5.20 ± 0.12 |

^1^Data are means ± SEM.
